# Metabolic syndrome in central Brazil: prevalence and correlates in the adult population

**DOI:** 10.1186/1758-5996-4-20

**Published:** 2012-05-14

**Authors:** Eliane Said Dutra, Kênia MaraBaiocchi de Carvalho, Édina Miyazaki, Edgar Merchán- Hamann, Marina Kiyomi Ito

**Affiliations:** 1 Programa de Pós-graduação em Ciências da Saúde, Faculdade de Ciências da Saúde, Universidade de Brasília, Campus Darcy Ribeiro, Brasília, DF, 70910-900, Brazil; 2 Programa de Pós-graduação em Nutrição Humana, Faculdade de Ciências da Saúde, Universidade de Brasília, Brasília, DF, 70910-900, Brazil; 3 Departamento de Estatística, Universidade de Brasília, Brasília, DF, 70910-900, Brazil; 4 Departamento de Saúde Coletiva, Faculdade de Ciências da Saúde, Universidade de Brasília, Brasília, DF, 70910-900, Brazil

**Keywords:** Metabolic syndrome, Prevalence, Developing country, Education, Brazil

## Abstract

**Background:**

The prevalence of metabolic syndrome (MetS) has increased in developing countries in recent decades. This syndrome, a clustering of metabolic abnormalities, has been correlated to various socioeconomic and behavioral variables. We investigated the prevalence and prevalence ratios (PR) of MetS and related factors in an adult population of the Federal District (FD) of Brazil, which is located in the central region of the country.

**Methods:**

A cross-sectional, population-based study conducted in 2007, with 2130 adults (aged 18 years or older) in the FD of Brazil. Metabolic syndrome was defined according to the recently harmonized criteria. The prevalence of MetS and PR were estimated for each sex according to the diagnostic components and the overall contribution of the selected correlates.

**Results:**

The overall prevalence of MetS was 32.0% (95%CI: 28.9–35.2), with no gender difference. The single component with the greatest contribution to the diagnosis of MetS was hypertension in men (PR 5.10, 95%CI: 3.17–8.22) and high waist circumference in women (PR 5.02, 95%CI: 3.77–6.69). The prevalence of MetS increased significantly and progressively with age and excess weight. In women, higher education was protective against MetS (PR 0.66, 95%CI: 0.49–0.89) compared to 8 or less years of education. There was no association between the prevalence of MetS and behavioral variables studied.

**Conclusions:**

This study provides comprehensive and alarming data about the prevalence of MetS among the adult population of Brazil’s FD. The results suggest that reducing education inequalities may be an important public policy goal to improve health outcomes, especially among women.

## Background

The burden of chronic non-communicable diseases (NCD) in developing countries continues to rise, with high morbidity and mortality due to cardiovascular diseases (CVD) [1,2]. Metabolic syndrome (MetS) is a complex disorder characterized by the association of cardiovascular risk factors and insulin resistance. The factors that define MetS include central obesity, dyslipidemia, hypertension and altered glucose metabolism [3]. The risk associated with CVD and type 2 diabetes increases as a cluster [4-6] or in accordance with individual components of MetS [7,8]. While debate continues over the definition of MetS, its clinical significance and value as an independent risk marker for cardiovascular disease (CVD) remains high [9,10].

Likewise, the public health relevance of MetS for countries in which economic resources are limited is unquestionable. Government policies to prevent CVD may be optimized when aimed to prevent and treat MetS as a whole rather than its individual risk factors [11].

Globally, the overall prevalence of MetS may vary according to the population, gender, age, geographic location, other correlated variables and the diagnostic criteria used [12,13]. In addition, its prevalence has increased in developing countries in recent decades [11]. The Cardiovascular Risk Factor Multiple Evaluation in Latin America (CARMELA) study included an assessment of MetS in seven urban Hispanic Latin American adult populations. The prevalence of MetS, defined according to the National Cholesterol Education Program Adult Treatment Panel III – NCEP/ATP III [14], was highest among CARMELA cities in Mexico City (27%) and lowest in Quito (14%) [15].

Underprivileged urban populations from developing countries have been shown to have an increased prevalence of overweight [16] and other risk factors for CVD [11]. Brazil is a non-Hispanic middle income country, and CVD is the leading cause of mortality there. Low income segments of the population are the most affected, especially with respect to premature deaths attributable to CVD [17]. In the few population studies conducted in Brazil, MetS prevalence ranged from 19% [18] to 25% [19] in urban populations. Among socioeconomic factors, educational inequality has consistently been associated with an increased risk of MetS in different populations [11,20,21], but this association has not been demonstrated for the South American population.

Recently, to simplify the diagnostic process and allow for comparison between countries, a unifying world-wide consensus definition for MetS was proposed [3]. According to the harmonized criteria, population and country-specific definitions for waist circumference thresholds should be used.

The aim of this study was to use the new consensus definitions to estimate the prevalence and prevalence ratios (PR) of MetS in the adult population of Brazil’s Federal District (FD), which includes the capital city Brasilia. Furthermore, the study sought to investigate the associated correlates for MetS prevalence in a sample with a wider age range.

## Methods

Brazil’s FD is located in the center of the country, with a population of 2,434,033 inhabitants, 68.9% of whom are 18 years or older [22]. The population has a diverse background, including people who were born locally and those who came from other parts of the country during the 50 years of the FD’s existence. This population-based household cross-sectional study representative of the population aged 18 years or older residing in the FD was conducted in 2007 to investigate the prevalence and risk factors associated with NCD. The study used a complex sampling plan with a multistage probability cluster design and with random sampling of census sectors, households and one adult per household in urban and rural areas of the FD [22]. The sample loss was considered to be 20%. Pregnant women and adults unable to understand or respond independently were excluded from the survey. Of the 2,726 adults that comprised the study sample, 2,130 individuals constituted the final sample that had anthropometric measurements taken and biochemical tests performed.

The study protocol was approved by the University of Brasilia’s Faculty of Health Sciences Research Ethics Committee, and informed consent was obtained from each participant. Household questionnaires were administered in face-to-face interviews. Interviewers were trained health sciences students or professionals. Reproducibility tests were conducted to guarantee reliable instrument and interviewer performance.

Anthropometric measurements were obtained twice with the participant in light clothing and barefoot. Body weight was measured using a digital scale, and height was determined by a portable stadiometer for body mass index (BMI) calculation (kg/m^2^). Waist circumference (WC) was measured to the nearest millimeter and taken midway between the lower limit of the rib cage and the iliac crest using a non-stretchable nylon tape. Arterial blood pressure readings were measured twice (15 min interval) in a seated position with an automatic apparatus (Omron, model HEM-705CP, USA), and the mean of the two values was calculated.

For biochemical tests, 12 h overnight fasting blood samples were assayed by standard methods for glucose, total cholesterol (TC), high-density lipoprotein cholesterol (HDLc) and triglycerides (TG) using an automatic biochemical analyzer Konelab 60i (Thermo Electron Co., USA). Low-density lipoprotein cholesterol (LDLc) was calculated by the Friedewald equation, excluding samples with triglyceride values > 400 mg/dL [23].

This study used the harmonized MetS criteria [3]. Metabolic syndrome (the dependent variable) was considered present when at least three of the following characteristics were observed: TG ≥ 150 mg/dL; HDLc < 40 mg/dL in men or < 50 mg/dL in women; systolic arterial blood pressure ≥ 130 mmHg and/or diastolic arterial blood pressure ≥ 85 mmHg or anti-hypertensive treatment; and glucose ≥ 100 mg/dL or anti-diabetic treatment. For abdominal obesity, the consensus calls for the “population- and country- specific definition” of cut points for elevated WC. No WC cut point of risk for MetS has been established for the Brazilian population as a whole. As Brazilians are predominantly of European ancestry [24], we adopted WC threshold values of ≥ 102 cm for men and ≥ 88 cm for women, one of the criteria recommended by the harmonized consensus based on American Heart Association/National Heart, Lung and, Blood Institute values for people of European origin [3].

The independent variables included socio-demographic, behavioral, nutritional and health status. Sociodemographic variables included age, sex, education, *per capita* income (legal minimal wage (MW) at the time of the field work ≈ US$150.00), time of residence in FD, marital status and self-reported skin color. Behavioral variables were fruit and vegetable intake, use of table salt, alcohol consumption and smoking status. Physical activity level was assessed by the International Physical Activity Questionnaire short version [25] and categorized into sufficiently active, insufficiently active and inactive [26]. Aside from MetS defining factors, the additional nutritional and health variables were BMI, TC and LDLc.

### Statistical analysis

To estimate the prevalence of MetS and associated factors, weighting factors were used so that a probability of being sampled was assigned to each participant. This procedure enabled the results to represent the adult population of FD according to sex, age and education level based on the official census data available at the time of analysis [21].

The database was built by double typing. Sample weights and the effect of complex sample design on the standard errors were treated using the survey command of STATA software version 9.2 (Stata Corp., College Station, Texas, USA). Initially, the prevalence of MetS and the number of MetS-defining components were calculated as well as the socio-demographic variable distributions with 95% confidence intervals (CI). Next, two multivariate models were tested. In the first model, the prevalence ratios (PR) between each diagnostic component and the MetS diagnosis with 95% CI were calculated to analyze the strength of the association between each MetS-defining component and diagnosis. The second model analyzed the overall contribution of all the independent variables to MetS diagnosis excluding the MetS-defining variables. In this model, after the initial crude analysis, the variables with a significant association with MetS were further adjusted. All the multivariate analyses were carried out using a Poisson regression with robust variance (log-linear) for each sex. We used the Poisson regression because it provides a better estimate of the prevalence ratios, which in turn represent a more meaningful effects measure for cross-sectional studies [27]. The significance level was set at *P*-value < 0.05.

## Results

The overall prevalence of MetS was 32.0%, with no difference observed between men (30.9%, 95%CI: 26.1–35.6) and women (33.0%, 95%CI: 29.5–36.6). According to the number of diagnostic components of MetS, only 13.6% had zero components, and 5.2% had all five components (Table1). The majority of subjects in the study were female, younger than 45 years old, with a *per capita* income below one minimum wage, married or living with a partner, and 50% had up to 8 years of schooling. The most frequently cited self-reported skin color category was “mixed” (44.8%).

**Table 1:** **Prevalence and distribution of sociodemographic variables (%), metabolic syndrome (MetS) and number of its components, among adults aged 18 years or older**^**a**^**(Federal District, Brazil, 2007)**

	**Total (n=2130)**		**Male (n = 586)**		**Female (n = 1544)**	
**Variable**	**%**	**95%CI**	**%**	**95%CI**	**%**	**95%CI**
MetS						
No	68.0	(64.8 – 71.1)	69.1	(64.4 – 73.9)	67.0	(63.4 – 70.5)
Yes	32.0	(28.9 – 35.2)	30.9	(26.1 – 35.6)	33.0	(29.5 – 36.6)
MetS components number						
0	13.6	(11.2 – 159)	14.8	(10.3 – 19.2)	12.5	(10.0 – 15.0)
1	31.8	(28.6 – 35.1)	31.2	(25.5 – 36.9)	32.4	(28.0 – 36.8)
2	22.6	(19.9 – 25.2)	23.2	(19.1 – 27.2)	22.0	(18.9 – 25.2)
3	15.7	(13.6 – 17.8)	15.2	(12.0 – 18.4)	16.1	(13.8 – 18.5)
4	11.1	(9.3 – 12.8)	10.5	(8.0 – 13.1)	11.6	(9.4 – 13.7)
5	5.2	(4.0 – 6.5)	5.1	(3.0 – 7.2)	5.3	(4.1 – 6.6)
Age (years)						
18–24	24.8	(20.9 – 28.8)	25.3	(18.2 – 32.3)	24.5	(21.2 – 27.7)
25–34	28.6	(24.5 – 32.7)	28.9	(20.8 – 37.1)	28.3	(24.4 – 32.2)
35–44	21.1	(18.4 – 23.8)	20.8	(16.5 – 25.2)	21.3	(18.8 – 23.7)
45–54	13.4	(11.8 – 15.1)	13.3	(10.4 – 16.2)	13.5	(11.5 – 15.5)
55–64	7.1	(5.9 – 8.2)	7.2	(5.4 – 9.0)	7.0	(5.8 – 8.2)
≥65	5.0	(3.5 – 6.5)	4.5	(2.8 – 6.2)	5.5	(4.0 – 6.9)
Education (years)						
0–8	50.1	(42.8 – 57.5)	51.9	(43.3 – 60.5)	48.6	(41.3 – 55.8)
9–11	31.3	(27.5 – 35.2)	29.3	(24.6 – 34.1)	33.1	(28.9 – 37.3)
≥ 12	18.5	(10.3 – 26.8)	18.8	(10.0 – 27.6)	18.3	(10.0 – 26.6)
*Per capita* income (minimal wages)						
< 0.5	31.6	(24.3 – 38.9)	23.9	(15.5 – 32.2)	38.4	(29.9 – 47.0)
0.5–1.0	25.1	(20.3 – 29.9)	25.1	(17.8 – 32.4)	25.2	(20.6 – 29.8)
> 1.0	43.3	(33.9 – 52.6)	51.1	(40.6 – 61.6)	36.3	(26.3 – 46.4)
Marital status						
Single	35.0	(30.8 – 39.2)	40.0	(32.7 – 47.3)	30.6	(26.5 – 34.6)
Married/living with partner	56.4	(52.4 – 60.4)	54.6	(47.1 – 62.1)	58.0	(53.3 – 62.6)
Widowed/separated	8.6	(6.9 – 10.3)	5.4	(2.7 – 8.0)	11.4	(9.3 – 13.6)
Skin color						
Black	14.1	(11.1 – 17.1)	15.6	(10.7 – 20.5)	12.1	(7.9 – 16.4)
Mixed (“pardo”)	44.8	(38.6 – 51.0)	47.2	(37.8 – 56.6)	42.7	(37.1 – 48.3)
White	30.3	(23.5 – 37.2)	28.0	(18.9 – 37.2)	32.3	(26.3 – 38.3)
Others	10.8	(6.3 – 15.2)	9.2	(3.7 – 14.7)	12.1	(7.9 – 16.4)

^a^ Weighted to represent distributions of population aged 18 years or older, according to gender, age and education based on Brazilian national census for the year 2000.

Table2 summarizes the contribution of individual diagnostic components to the observed PR for MetS. The single crude component that contributed most to the diagnosis of MetS was hypertension or elevated blood pressure in men (PR 11.01, 95%CI: 6.41–18.93) and high WC in women (PR 11.26, CI95%: 8.55–14.83). Adjustment for all diagnostic components did not change these results, and a high WC led to a greater than five-fold increase in the prevalence of MetS among women (PR 5.02, CI95%: 3.77–6.69). The other two most important components that increased MetS prevalence were hypertriglyceridemia and low HDLc in men and low HDLc and elevated blood pressure in women.

**Table 2:** **Crude and adjusted prevalence ratios of metabolic syndrome according to diagnostic components among adults aged 18 years or** older ^a^**, by gender (Federal District, Brazil, 2007)**

	**Prevalence ratio (95%CI)**							
	**Male**				**Female**			
	**Crude**		**Adjusted^b^**		**Crude**		**Adjusted^b^**	
Elevated blood pressure								
No	1		1		1		1	
Yes	11.01	(6.41 – 18.93)	5.10	(3.17 – 8.22)	6.29	(4.96 – 7.98)	2.45	(2.02 – 2.97)
Elevated fasting glucose								
No	1		1		1		1	
Yes	4.62	(3.52 – 6.05)	1.80	(1.47 – 2.20)	3.98	(3.40 – 4.65)	1.53	(1.36 – 1.72)
Elevated waist circunference								
No	1		1		1		1	
Yes	4.92	(3.81 – 6.36)	1.63	(1.30 – 2.03)	11.26	(8.55 – 14.83)	5.02	(3.77 – 6.69)
Elevated triglycerides								
No	1		1		1		1	
Yes	6.78	(4.28 – 10.72)	3.03	(2.26 – 4.86)	4.32	(3.64 – 5.13)	1.84	(1.63 – 2.08)
Low HDLc								
No	1		1		1		1	
Yes	5.82	(3.49 – 9.71)	2.97	(1.95 – 4.51)	4.63	(3.32 – 6.44)	2.71	(2.14 – 3.45)

^a^ Weighted to represent distributions of population aged 18 years or older, according to gender, age and education based on Brazilian national census for the year 2000.

^b^ Adjusted for all other MetS diagnostic components.

Tables 3 and 4 present PR according to sociodemographic, behavioral and selected health variables. In the crude PR analysis, the following variables did not show significance and were excluded: education, *per capita* income, skin color, fruit and vegetable intake, extra table salt, alcohol consumption, smoking and LDLc levels (for males); and *per capita* income, skin color, fruit and vegetable intake and current physical activity level (for females; data not shown). Subsequently, in the multivariate analysis, the first adjustment was performed for age. Among men (Table3), variables that showed significance at this level were age, BMI and TC. For women (Table4), age, education, marital status, alcohol consumption, BMI and TC were significantly associated with MetS. 

**Table 3:** **Prevalence (%), crude and adjusted prevalence ratios of metabolic syndrome among male adults aged 18 years or** older ^a^**, by selected sociodemographic, behavioral, nutritional and health status variables (Federal District, Brazil, 2007)**

	**Prevalence (95%CI)**	**Prevalence ratio (95%CI)**		
		**Crude**	**Adjusted^b^**	**Adjusted^c^**
Age (years)				
18–24	7.3 (0.0 – 14.8)	1	1	1
25–34	24.4 (16.0 – 32.7)	3.35 (1.27 – 8.88)	3.35 (1.27 – 8.88)	2.47 (1.15 – 5.33)
35–44	35.4 (26.3 – 44.4)	4.87 (1.94 – 12.2)	4.87 (1.94 – 12.2)	3.06 (1.44 – 6.51)
45–54	55.3 (44.2 – 66.4)	7.60 (3.08 – 18.7)	7.60 (3.08 – 18.7)	3.70 (1.73 – 7.87)
55–64	65.1 (50.5 – 79.7)	8.95 (3.64 – 22.0)	8.95 (3.64 – 22.0)	4.17 (1.96 – 8.86)
≥ 65	57.4 (41.7 – 73.1)	7.89 (3.14 – 19.8)	7.89 (3.14 – 19.8)	4.25 (1.97 – 9.17)
Residence time in Federal District (years)				
< 10	20.8 (9.9 – 31.8)	1	1	
10–19	24.8 (15.8 – 33.8)	1.19 (0.63 – 2.25)	1.17 (0.65 – 2.10)	
20–29	26.6 (16.6 – 36.6)	1.28 (0.70 – 2.33)	1.17 (0.68 – 2.00)	
≥30	45.5 (38.6 – 52.4)	2.19 (1.30 – 3.67)	1.16 (0.70 – 1.94)	
Marital status				
Single	14.8 (8.7 – 20.9)	1	1	
Married/living with partner	41.4 (35.0 – 47.7)	2.79 (1.74 – 4.47)	1.36 (0.78 – 2.35)	
Widowed/separated	43.4 (16.4 – 70.4)	2.93 (1.47 – 5.84)	1.21 (0.62 – 2.37)	
Current physical activity level				
Active	26.3 (20.5 – 32.2)	1	1	
Insufficiently active	35.6 (22.2 – 49.0)	1.35 (0.90 – 2.03)	1.24 (0.86 – 1.79)	
Inactive	41.8 (29.9 – 53.6)	1.58 (1.13 – 2.22)	1.23 (0.92 – 1.63)	
Body mass index (kg/m^2^)				
Normal (<25)	6.4 (3.3 – 9.4)	1	1	1
Overweight(25–29)	38.4 (31.8 – 44.9)	6.03 (3.52 – 10.3)	4.52 (2.64 – 7.74)	4.40 (2.57 – 7.55)
Obese (≥30)	84.9 (73.7 – 96.1)	13.3 (8.05 – 22.1)	9.99 (5.92 – 16.9)	9.77 (5.79 – 16.5)
Total cholesterol (mg/dL)				
<200	23.4 (17.2 – 29.6)	1	1	1
≥200	42.9 (34.6 – 51.1)	1.83 (1.32 – 2.54)	1.43 (1.08 – 1.91)	1.14 (0.91 – 1.42)

^a^ Weighted to represent distributions of population aged 18 years or older, according to gender, age and education based on Brazilian national census for the year 2000.

^b^ Adjusted for age.

^c^ Adjusted for age, body mass index and total cholesterol.

**Table 4:** **Prevalence (%), crude and adjusted prevalence ratios of metabolic syndrome among female adults aged 18 years or** older ^a^**, by selected sociodemographic, behavioral, nutritional and health status variables (Federal District, Brazil, 2007)**

	**Prevalence (95%CI)**	**Prevalence ratio (95%CI)**		
		**Crude**	**Adjusted^b^**	**Adjusted^c^**
Age (years)				
18–24	10.3 (3.7 – 17.0)	1	1	1
25–34	21.3 (16.0 – 26.5)	2.06 (1.13 – 3.74)	2.06 (1.13 – 3.74)	1.67 (0.98 – 2.83)
35–44	37.4 (31.6 – 43.2)	3.62 (2.04 – 6.44)	3.62 (2.04 – 6.44)	2.18 (1.31 – 3.66)
45–54	56.0 (47.2 – 64.9)	5.42 (3.08 – 9.55)	5.42 (3.08 – 9.55)	2.72 (1.61 – 4.60)
55–64	67.9 (59.7 – 76.1)	6.57 (3.74 – 11.55)	6.57 (3.74 – 11.55)	2.88 (1.69 – 4.90)
≥ 65	77.0 (68.4 – 85.5)	7.45 (4.23 – 13.11)	7.45 (4.23 – 13.11)	3.31 (1.90 – 5.75)
Education (years)				
0–8	43.5 (37.2 – 49.7)	1	1	1
9–11	24.7 (20.5 – 29.0)	0.57 (0.46 – 0.70)	0.78 (0.64 – 0.96)	0.87 (0.73 – 1.04)
≥ 12	20.5 (14.4 – 26.6)	0.47 (0.33 – 0.67)	0.51 (0.37 – 0.70)	0.66 (0.49 – 0.89)
Residence time in Federal District (years)				
< 10	21.9 (14.7 – 29.1)	1	1	
10–19	28.8 (23.1 – 34.5)	1.31 (0.93 – 1.85)	1.14 (0.83 – 1.57)	
20–29	28.8 (23.1 – 34.5)	1.31 (0.93 – 1.85)	1.14 (0.83 – 1.57)	
≥30	49.7 (43.8 – 55.7)	2.27 (1.70 – 3.03)	1.10 (0.82 – 1.47)	
Marital status				
Single	20.3 (15.5 – 25.2)	1	1	1
Married/living with partner	34.8 (30.0 – 39.6)	1.71 (1.32 – 2.22)	1.22 (0.95 – 1.57)	0.95 (0.77 – 1.17)
Widowed/separated	58.3 (50.4 – 66.1)	2.87 (2.20 – 3.74)	1.28 (0.99 – 1.66)	1.03 (0.83 – 1.29)
Extra table salt				
No	35.0 (30.8 – 39.2)	1	1	
Yes	28.0 (20.7 – 35.3)	0.80 (0.64 – 1.00)	0.92 (0.75 – 1.12)	
Alcohol consumptiong				
Never	36.8 (32.7 – 40.8)	1	1	1
No abusive	23.8 (15.8 – 31.9)	0.65 (0.49 – 0.86)	0.74 (0.55 – 0.99)	0.90 (0.69 – 1.17)
Abusive	21.7 (10.4 – 33.0)	0.59 (0.36 – 0.97)	0.75 (0.50 – 1.13)^e^	0.71 (0.48 – 1.04)
Smoking				
Never	29.4 (25.7 – 33.1)	1	1	
Former smokers	55.0 (45.0 – 65.0)	1.87 (1.54 – 2.28)	1.41 (1.18 – 1.67)	
Current smokers	34.2 (23.7 – 44.7)	1.16 (0.88 – 1.53)	1.12 (0.86 – 1.46)	
Body mass index (kg/m^2^)				
Normal (<25)	8.4 (6.1 – 10.6)	1	1	1
Overweight(25–29)	45.5 (38.3 – 52.8)	5.45 (4.13 – 7.20)	4.29 (3.21 – 5.75)	4.09 (3.07 – 5.46)
Obese (≥30)	78.1 (72.6 – 83.5)	9.34 (7.20 – 12.1)	7.04 (5.32 – 9.31)	6.78 (5.15 – 8.92)
Total cholesterol (mg/dL)				
<200	25.5 (20.7 – 30.3)	1	1	1
≥200	46.1 (40.7 – 51.6)	1.81 (1.52 – 2.16)	1.21 (1.02 – 1.43)	1.20 (1.05 – 1.36)
LDLc (mg/dL)				
<130	28.1 (23.7 – 32.5)	1	1	
130–159	36.3 (30.2 – 42.4)	1.29 (1.05 – 1.58)	0.99 (0.82 – 1.19)	
≥160	52.3 (43.9 – 60.7)	1.86 (1.49 – 2.33)	1.09 (0.90 – 1.32)	

^a^ Weighted to represent distributions of population aged 18 years or older, according to gender, age and education based on Brazilian national census for the year 2000.

^b^ Adjusted for age.

^c^ Adjusted for age, education, marital status, alcohol consumption, body mass index and total cholesterol.

After the final adjustment, the prevalence of MetS increased significantly and progressively with age (*P* < 0.001) and BMI ≥ 25 kg/m^2^ (*P* <0.001) in both men and women. For women (Table4), there was a 20% increase in the frequency of MetS in those with total cholesterol levels greater than or equal to 200 mg/dL (*P* <0.05). Only in women, higher education (12 or more years of study) was protective against MetS (PR 0.66, 95% CI: 0.49–0.89). Younger women tended to have more than 12 years of education when compared to older women (results not shown). No association was observed between behavioral variables and the prevalence of MetS.

## Discussion

To our knowledge, this is the first published population-based study that determined the prevalence of MetS in Central Brazil. The results indicate that the population of the FD is at significant risk of morbidity and mortality from CVD and type 2 diabetes due to the high prevalence of MetS (32.0%). This disturbing result is not surprising because trends of excess weight among Brazilians [28] and the country’s surveillance system have pointed to the increasing prevalence of excess weight and other factors associated with cardiovascular risk in our adult population [29]. Our results reinforce an alarming public health trend [16,17].

The overall prevalence of MetS found in our study is similar to that in the US population (34.0%) reported by the 2003–2006 National Health and Nutrition Examination Survey (NHANES) [30]. Compared to other middle income countries, our result is similar to Venezuela, with 31.2%, [31] greater than the 26.9% described for Peru [32], and lower than the 41.4% reported in India [33]. Apart from methodological differences between studies, variability in the prevalence of MetS between populations could be explained by demographic, epidemiological and nutritional transitions [34], as well as environmental and social influences [35], and ethnic differences [36]. As for the prevalence of MetS compared to other Brazilian studies, the results of this study may reflect the differences in the statistical analyses [18,19] and fasting glucose cut-off points [18]. Additionally, regional differences and the period of data collection (for example the early 2000s) are factors that deserve consideration.

There is a gradient of risk for CVD with increasing number of MetS components [37,38]. The identification of persons with zero diagnostic criteria of MetS, a group that comprised 13.6% of our study population, is clinically relevant as these people seem to have a substantially reduced risk of developing CVD [39]. Those without any risk should be an important target for the identification of the beneficial factors associated with protection against MetS.

The difference between genders in which three components most strongly accounted for the diagnosis of MetS in our sample was unexpected (Table2). The practical utility of this information is the knowledge that hypertension in men and abdominal obesity in women could be the single best predictors of MetS related co-morbidity for screening purposes.

Although the World Health Organization [40] identified environmental factors such as smoking, low intake of fruit and vegetables, physical inactivity, and other factors as important contributors to a large proportion of mortality attributed to CVD, no association was observed between behavioral correlates and MetS in the present study (Tables 3 and 4). Similar results were described in a recent cohort study for MetS in Portugal [41], and these results point to the complex nature of MetS-associated factors.

The strong association between abdominal obesity and MetS is well-recognized [42,43]. In the unified statement on the definition of MetS, the WC is the only parameter to be evaluated according to population and country-specific definitions [3]. The unified consensus suggests WC ≥ 90 cm for male and ≥ 80 cm for female as thresholds for abdominal obesity among ethnic South and Central Americans, derived from Asian population studies [44]. However, the assignment of these cutoff points does not fit the ethnic characteristics of the entire population living in these regions [45], such as the Brazilian population. Brazil has a highly heterogeneous population, with inter- ethnic admixtures of people from European, African and indigenous backgrounds [46]. A recent study on the genomic ancestry of different geographical regions of Brazil has ratified the predominance of European ancestry among Brazilians [24]. Thus, the use of ≥102 cm for men and ≥ 88 cm for women as WC cut points in our study was a pragmatic decision based on the above rationale, as recognized in the 2009 harmonized consensus [3]. Further studies are needed to define the WC cut points representative of the Brazilian population.

Our study identified education as a protective factor against MetS in women (PR 0.66, 95% CI: 0.49–0.89), which is in agreement with other studies [21,36,47]. Analyzing the age category (results not shown), women with 12 or more years of schooling tended to be in the younger categories. The increased number of women with 12 or more years of education is a recent phenomenon in Brazil [48], and it may partly explain the observed result. Among South Koreans, women with less education tended to be in the older birth cohorts and had a higher prevalence of MetS [47]. In women, the cumulative effect of poor socioeconomic status during childhood and adulthood, including education, seems to influence the risk of MetS [49]. An assessment of potential mediators of educational inequalities in our population in the context of the transition process in developing countries and how they affect men and women differently could identify the best exponents of this phenomenon.

Likely limitations of our study are related to the nature of cross-sectional studies, including the potential for survival bias and temporal ambiguity. Considering that diagnostic factors and other correlates that constitute MetS may fluctuate over time, the diagnostic accuracy may increase with subsequent data acquisition. In our sample, women were clearly over-represented, but we minimized this effect by applying adjustment methods. The strengths of this study lie in the probabilistic sampling and the use of prevalence ratios in the multivariate analyses, making these results a sound basis for planning public health interventions.

## Conclusions

In conclusion, our study revealed that 32.0% of the adult population of Brazil’s FD had MetS in 2007. The components of this syndrome were gender-specific, and education was protective for women. The study enabled a sound measure of MetS for data comparison and suggests that reducing education inequalities may be an important public policy goal to improve health outcomes, especially among women.

## Competing interests

The authors declare that they have no competing interests.

## Authors’ contributions

ESD undertook execution, analysis and wrote the first draft of the manuscript. ESM contributed with statistical analyses. KMBC, EM and MKI contributed in the design, execution and critical discussion of the study. All authors read and approved the final manuscript.
